# Unaccompanied foreign minors: the Novara experience as a reference center of Eastern Piedmont (Italy)

**DOI:** 10.1007/s00414-025-03498-y

**Published:** 2025-04-30

**Authors:** Federica Collini, Samuele Baldi, Sarah Gino

**Affiliations:** 1 Department of Health Sciences, University of Eastern Piedmont, Novara, Italy; 2 A.O.U. “Maggiore della Carità”, Direzione Medica dei Presidi Ospedalieri, Novara, Italy

**Keywords:** Age estimation, Unaccompanied minors, Wrist RX, Bone age assessment, Migrants

## Abstract

**Background:**

20% of migrants who reach Italy are minors, often Unaccompanied Foreign Minors (UFM). If they are minors, they have the right to remain on EU territory, but in case of doubt, the authorities ask to ascertain their age. This is currently done through different procedures.

**Methods:**

The aim of this study was to understand the validity of the protocol adopted by the ‘Maggiore della Carità hospital in Novara (Italy), reference center for Eastern Piedmont, for ascertaining the age of self-proclaimed minors by comparing the results with those of other European realities. A cross-sectional study was conducted by analyzing the final reports of UFM examined at this hospital between January 1st, 2018, and December 31st, 2023. Socio-demographic, clinical, radiological and forensic data were collected.

**Results:**

Three hundred and one migrants were evaluated: 97% was male with an average age reported of 16.32 years ± 1.35. The estimated age by wrist X-ray was 17.81 ± 1.52. The physical examination of sexual maturation was not statistically correlated with the age estimated by X-rays (*r* = 0.36). No migrant was certified as an adult. Pathologies and injuries were frequently described.

**Conclusion:**

Comparing these results with those of similar European studies, it seems that the Piedmont protocol is unable to accurately estimate the age, even though it is one of the most complete Italian one. The effort to assign an age as close as possible to the actual one is a prerequisite for recognizing the rights of these individuals, but the method must achieve concrete and decisive results without violating their integrity.

## Introduction

For many years now, the medias have been informing daily about the constant increase in the number of migrants reaching the shores of the European Union [1], particularly the southern countries, including Italy (394,582 landings between 2018 and 2023) [2]. The social problems arose by this unprecedented phenomenon are numerous, but not everyone knows that it is also a medico-legal issue [3].

Indeed, 20% of the migrants who reach the Italian coasts are minors, and among these, 75% are not accompanied by relatives or parents: these are known as unaccompanied foreign minors (UFM) [4–6]. If these young people were really minors, they have the right to obtain specific reception and shelter regimes in Europe, but in case of doubt about their age, the competent authorities may request a medical age assessment to evaluate their actual rights.

This study deals with the delicate topic of age assessment for UFM, which has thus assumed fundamental importance in countries where the migratory flow has increased over the years. However, significant difficulties may arise in carrying out age evaluations, as a shared protocol for conducting them has not yet been established across Europe [7]: more often, especially in Italy, each region or even each healthcare facility follows different methods, resulting in a widely heterogeneous picture [8].

The Study Group on Forensic Age Diagnostics (AGFAD), established in Berlin in 2000, identified the following steps for age assessment in living individuals [9–11]:

1. Physical evaluation, including anthropometric measurements (height, weight, body type), and assessment of sexual maturation signs. In Italy the sexual character maturation is evaluated using the Tanner stages^1^. It is essential in this context to recognize or exclude any pathologies that may lead to alterations in physical development.
2. X-ray of the left hand-wrist district, evaluating the shape and size of the individual bone elements, as well as the ossification of the epiphyseal plates. This X-ray is interpreted in Italy using the Greulich and Pyle atlas [12– 17] and, more rarely, the Tanner-Whitehouse method [16, 18, 19]. If the hand development is complete, the X-ray or CT scan technique should be extended to the clavicle, as it is the last bone segment to complete ossification (beyond 21 years) [20, 21];
3. Examination of the dentition, both by clinical evaluation to observe tooth eruption, and by orthopantomography to evaluate the mineralization of the root apex [22–30].

The method proposed by AGFAD is not strictly followed in Italy, where age assessment in case of doubt about reaching adulthood has been required by judges since 1988 for criminal proceedings [31]. Regarding the age of migrants, since 2008, the national government has unsuccessfully attempted to issue an unified protocol. Eventually, Italian Law 47/2017 [32], and the Protocol of the Conference of Regions and Autonomous Provinces emanated in 2020 [33], established an assessment procedure that includes three steps to which the young migrants must be subjected:

1. An interview with a social worker, to put the examinee at ease and understand their migratory story.
2. An interview with a psychologist, to evaluate the psychical development of the migrant.
3. A pediatric examination, using the less invasive methods as possible and respectful of the presumed age, sex and psycho-physical integrity of the minor.

The involvement of a Medical Examiner is not foreseen, and the use of radiological techniques, considered too invasive, is reserved for exceptional cases.

However, it is evident that these three steps, being extremely subjective, cannot guarantee the accurate determination of age. For this reason, many regions, including Piedmont, have maintained their own protocols [34].

In Piedmont the local protocol used to assess UFM’s age since 2018, includes a pediatric examination, the left hand-wrist X-ray and, a final certificate released by the Medical Examiner, based on the previous two steps [35].

Once the pediatrician obtains the migrant’s consent, the medical history is collected, underlining in particular the presence of chronical illnesses that could have affected the regular physical development, in order to avoid any under or overstatement of the age. After that, a complete physical examination is issued, focusing on the erupted third molars and to the pubertal development with the Tanner’s scale. Therefore, the X-ray is carried out and the radiologists give their opinion on the bone age of the examinee, using the Greulich and Pyle’s atlas. Eventually, the Medical Examiner writes the final report, generally based on the bone age, with the expression a range of ± 2 years due to a variability among individuals and imposed by the protocol. The minimum age (with the “-2 range”) is taken into consideration for legal purposes.

## Materials and methods

In this cross-sectional study, all the reports of demographic assessments of unaccompanied foreign minors (UFM) issued at the “A.O.U. Maggiore della Carità” of Novara between January 1st, 2018, and December 31st, 2023, were reviewed, and the data collected was anonymized in an Excel database.

The following information were analyzed:

1. Sex.
2. Declared age at the date of the assessment, starting from the declared date of birth.
3. Country of origin.
4. Authority requesting the examination.
5. Reason for the request.
6. Estimated age at the end of the assessment, based on skeletal age, with margin of error.
7. Physical examination (with particular attention to injuries, scars, pathological situations).
8. Pubertal development stage according to Tanner and testicular volume.

A statistical analysis of these aspects was then carried out, evaluating:

- number of assessments per year and in total, correlated with the number of landings on Italian coasts and the estimate of UFM entries in the same period.
- absolute number and percentual frequency of males and females.
- absolute frequency of country of origin, requesting entity, reason for the request.
- mean, standard deviation, and box plot of the stated age, as well as the most probable age (grown out of X-ray), minimum and maximum age attributable based on the uncertainty range.
- average difference between the stated age and the age estimated as most probable.
- average of the Tanner stage and testicular volume found during the physical examination and correlation of the latter with the X-ray assessed age;
- estimation of the number of immigrants with pathological conditions, skin lesions, or distinctive signs such as scars or dental avulsions.

## Results

**Fig. 1:**
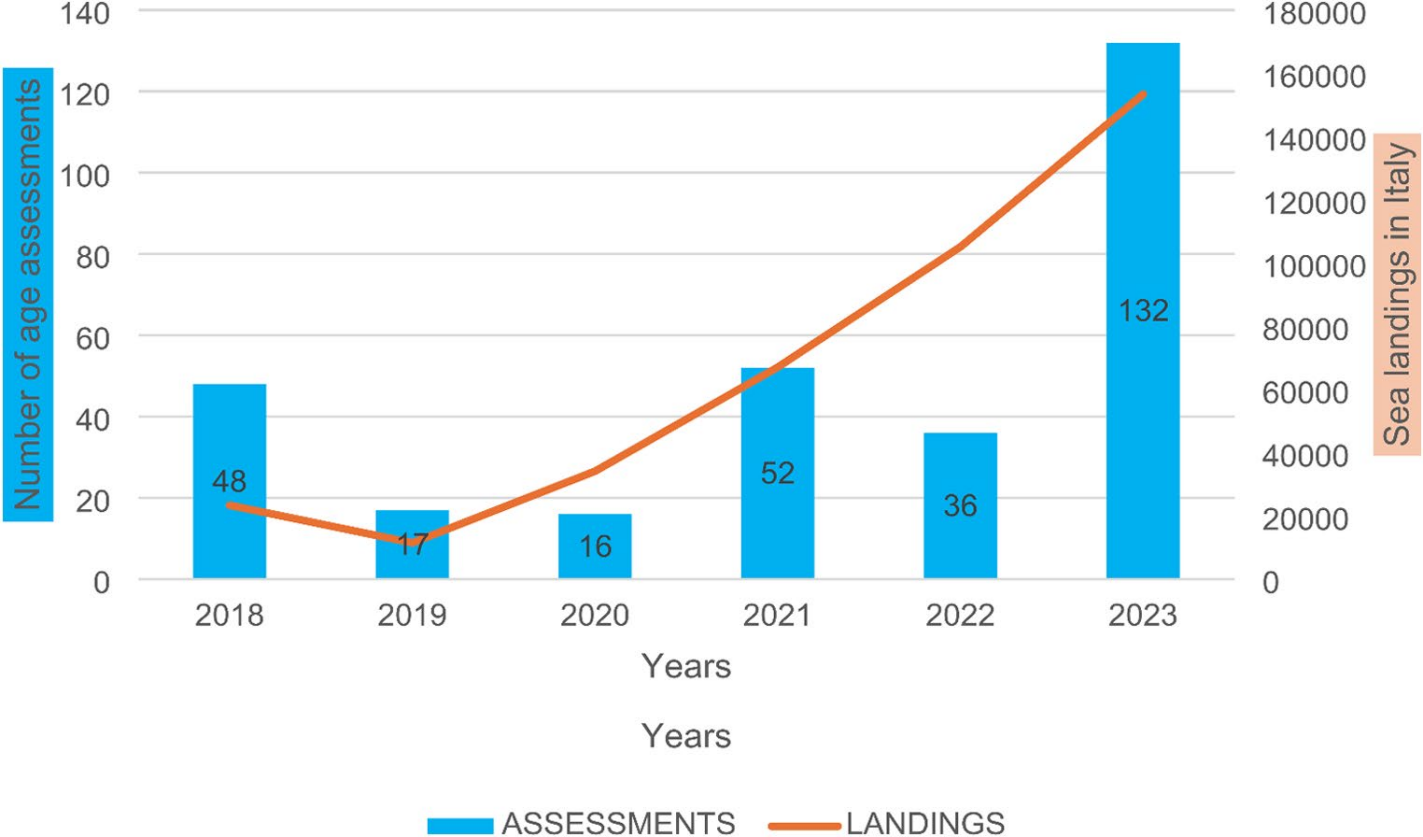
Number of UFM assessments conducted each year at the Novara Hospital from 2018 to 2023, in comparison with the annual number of landings by sea in Italy

During the period from January 1st, 2018, to December 31st, 2023, 301 age assessments of UFM were conducted at the University Hospital Center “Maggiore della Carità” hospital in Novara. The annual absolute frequency of the alleged minors during this period was highly variable: specifically, the assessments conducted decreased from 48 in 2018 to 16 in 2019 and 17 in 2020, while the year with the highest number of visits (132) was 2023 (Fig. 1).

**Fig. 2:**
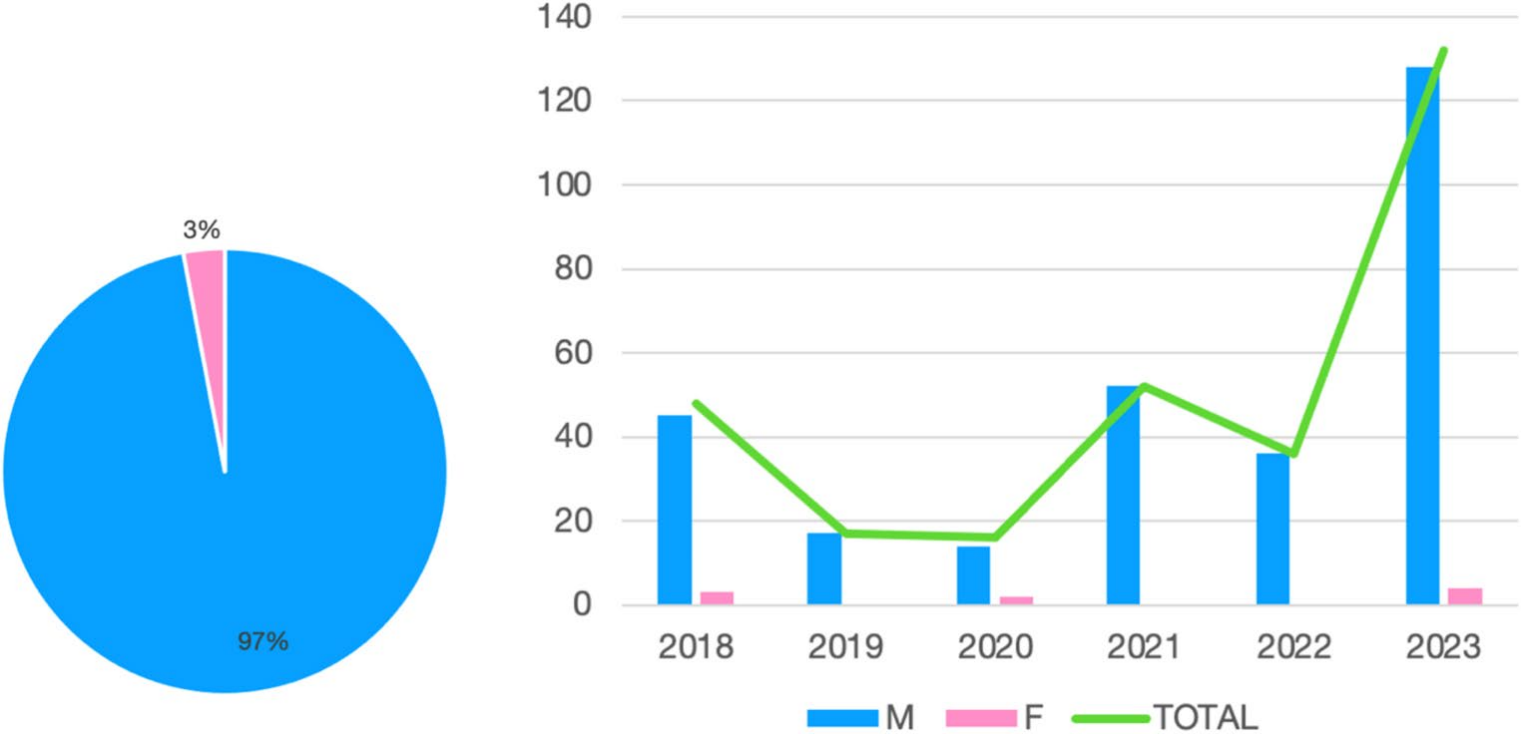
Gender of the UFM under examination. On the left, a pie chart of the total for the six years; on the right, a histogram by year

Figure 2 shows the distribution per gender of the migrants examined.

The nationalities declared by the examinee are (listed in descending order): Pakistan (41), Egypt (40), Gambia (36), Bangladesh (34), Tunisia (31), Guinea (21), Ivory Coast (16), Nigeria (10), Afghanistan (8), Cameroon (8), Senegal (8), Morocco (7), Turkey (7), Mali (6), Kosovo (5), Somalia (5), Sierra Leone (4), Benin (3), Burkina Faso (3), Eritrea (2), Chad (1), Ghana (1), India (1), Niger (1), Central African Republic (1), Togo (1).

Grouping these origins by geographical area, about 42% came from Sub-Saharan Africa, followed by the Indian subcontinent (28%) and the Maghreb (26%). Approximately 4% came from Europe (considering Kosovo and Turkey). The peak of alleged minors from Indian subcontinent countries was observed in 2020–2021 (63% and 71%). In 2022, the examination of young people from the Maghreb prevailed (58%), and in 2023 a significant number of exams were conducted on from Sub-Saharan Africa’s migrants (64% of the total).

**Fig. 3:**
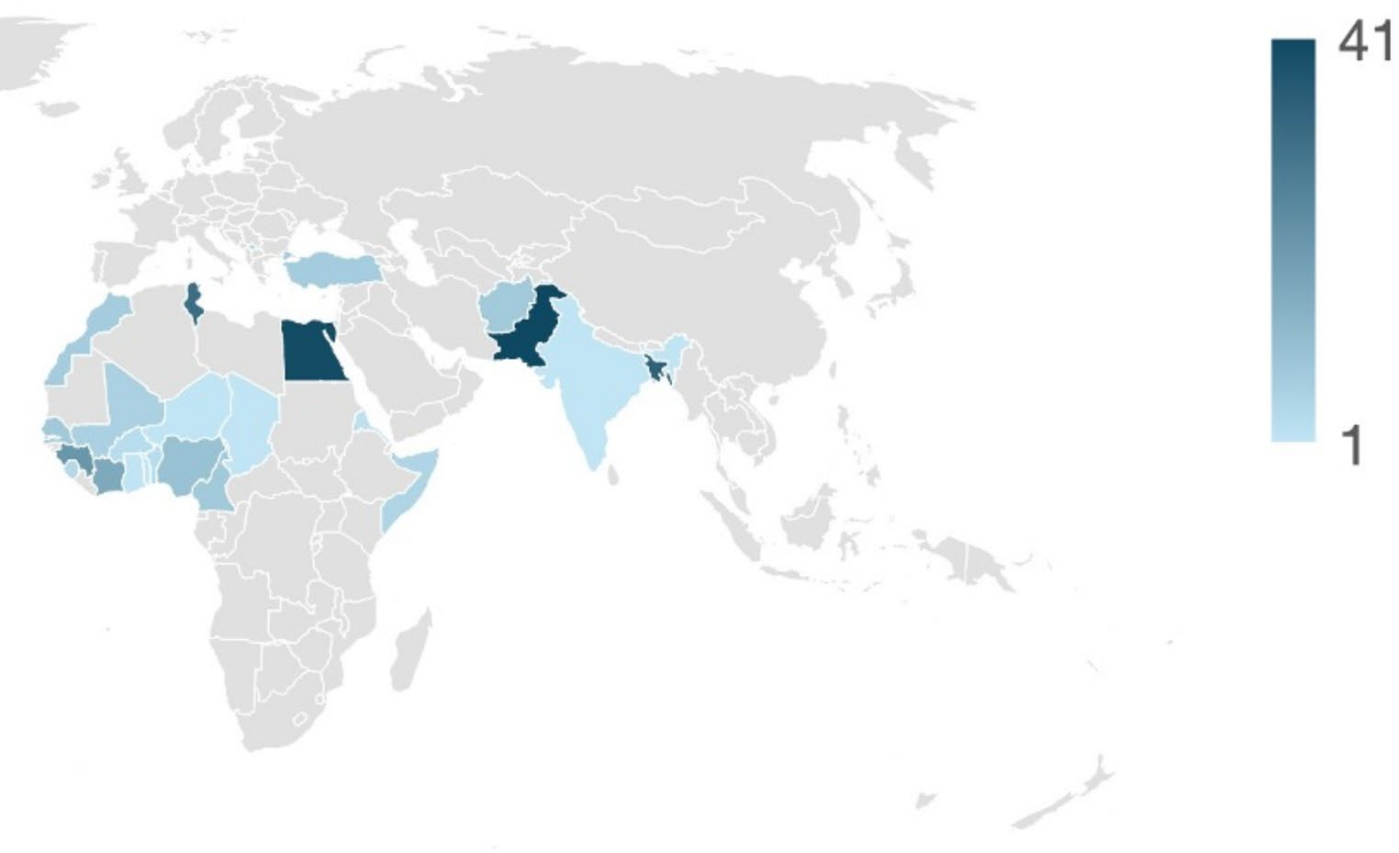
Map of the countries of origin of the 301 UFM included in the present study. Increasing color intensity based on increasing absolute frequency, as per legend

Focusing now on the referring entities, a total of 118 young people were sent by social services (39%), 92 by the Prosecutor’s Office (31%), 67 by minor communities (22%), 15 by Police Headquarters (5%), and 9 by the Police Officers (3%). In the early years examined, the request almost always came officially from the Prosecutor’s Office at the Juvenile Court of Turin (73% in 2018), while in recent years it has come from minor communities (64% in 2022), and from the social services of the municipality of residence (78% in 2023).

The number of minors sent for justice needs is also notable. In total, 10 out of 301 minors (just over 3%) were escorted to the hospital by police officers (4 in 2018, 5 in 2019, and 1 in 2020).

The entities requesting UFM assessments must state the reason for which the assessment is necessary. In this study, in 75% of cases the requests were due to the absence of documents necessary to prove the young person’s age (225 times). In a total of 58 times (19%) the assessment was carried out in “the minor’s interest”, in 11 for justice purposes (about 4%), and in 5 cases (less than 2%) to ensure international protection for the minor.

**Fig. 4:**
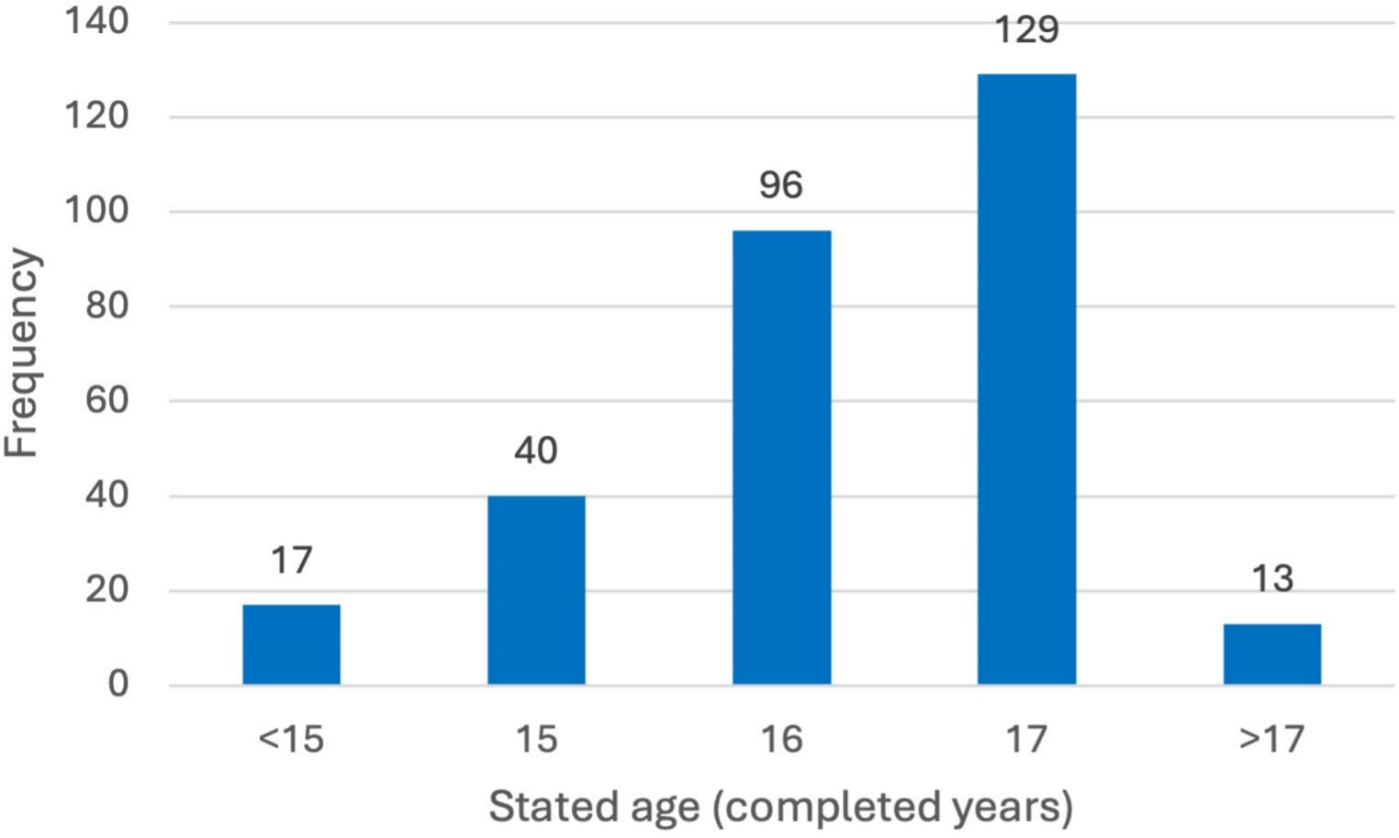
Histogram of the stated age of the alleged unaccompanied minors

Out of the 301 assessments performed, six were not considered in the study: one due to the loss of documentation and five because, in the first year of using the Piedmont regional protocol, the report had certified adulthood with certainty, without expressing a margin of error, making the assessment invalid by law. Of the 295 valid reports, the absolute frequencies of the age declared by each minor are summarized in the following graph (Fig. 4).

The average declared age was 16.32 years ± 1.35 (standard deviation).

The hand-wrist X-ray examination applied the Greulich-Pyle method 294 times and the Tanner-Whitehouse method once. The average age resulting from the X-ray, and thus certified, was 17.81 years ± 1.52. A bone age of 19 years old was found in 41.4% of cases, and in total, 71.5% (211 in total) of the X-rays showed an age older than or equal to 18. In all situations, the medical examiner confirmed the age resulting from the X-ray: in 237 cases (80.3%) an age older than the declared one was found, in 38 (12.9%) the same age, and in 20 cases (6.8%) a younger age.

**Fig. 5:**
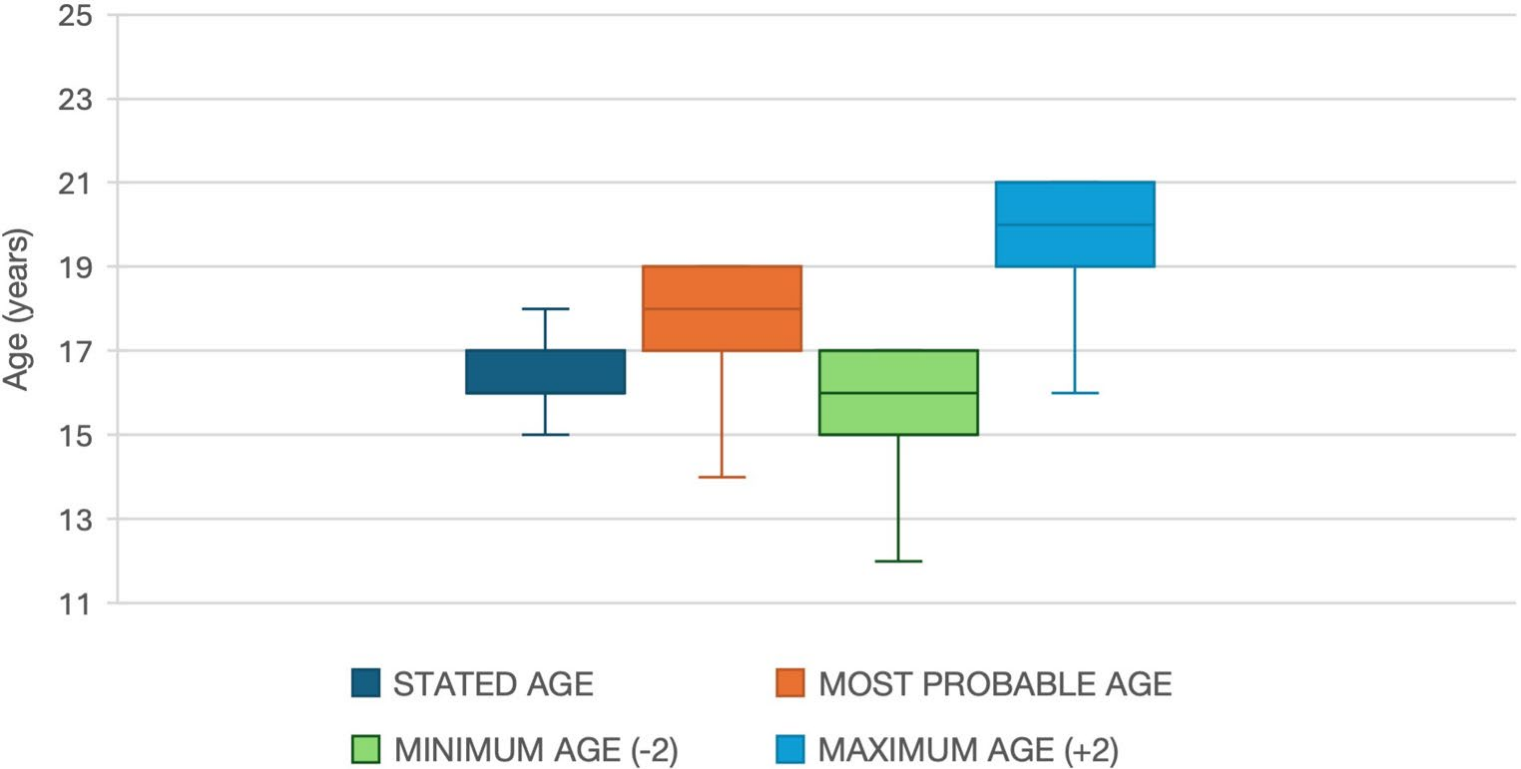
Box plot diagrams of the stated age, most probable age, minimum and maximum age (based on the range +/- 2 years imposed by the protocol)

Table 1 and Fig. 5 show the application of the “uncertainty range” to the most probable age, detected by X-ray examination.

**Table 1:** Mean of the age stated by migrants, most probable age found by X-ray examination, minimum and maximum age based on the empirical range  ±  2 years imposed by the protocol

Mean stated age	Mean detected age	Mean minimum age (empirical − 2 years)	Mean maximum age (empirical + 2 years)
16.32 ± 1.35	17.81 ± 1.52	15.81 ± 1.52	19.81 ± 1.52

Many additional pieces of information can be obtained from the pediatric examination. For the purpose of age assessment, an evaluation of sexual development using the Tanner staging was carried out in 187 males (out of 292) and in 4 females (out of 9). For males, the fifth stage was the most frequent found Tanner stage (in 163 patients), followed by the fourth (14), the third (8), and the second (2). The average testicular volume was 22.4 ml, (30 ml in 4 cases, 25 ml in 113, 20 ml in 44, 18 ml in 2, 15 ml in 18, 12 ml in 4, 8 ml in 2). No statistically significant correlation was found between testicular volume and age estimated from the wrist X-ray (Pearson’s *r* = 0.36).

The results for females were more uniform, with a diagnosis of the fifth Tanner stage in all cases analyzed.

The pediatric examination also proved essential for detecting any ongoing or past conditions to be treated, although generally infrequent. Among the most common disorders there were dermatological (three cases of scabies, one of Tinea Corporis, one of psoriasis) and urological (two confirmations of cryptorchidism, two of hydrocele, and one of hypospadias) ones, followed by infectious (one diagnosis of malaria, one of pneumonia) and cardiological findings (two murmurs from suspected mitral insufficiency).

At the oral cavity inspection, when performed, the hygiene levels of the migrants were found to be fair, with about ten cases of multiple and painful caries.

The general inspection is of great importance also for medico-legal purposes, so the pediatricians conducting the examination carefully described any lesions found during the physical examination, bringing them to the attention of the forensic pathologist.

In 24 cases healed injuries were observed keloids (3 patients), bone fractures (3 patients), 2 minors were treated for extensive burns, 3 declared they had been beaten so severely that they lost one or more teeth (in one case a dental prosthesis of the upper arch was noted), and in one case ankylosis of the upper limb was observed. One young person brought to the attention of the doctors cysts on the lower limb, later attributed to gunshot wounds. In some cases, more recent injuries incompatible with injuries sustained in the country of origin or during the journey were found (mainly orbital bruises and lacerations) (Table 2]).

**Table 2:** Summary of the most frequently observed injuries among the migrants examined

Type of injury	Number of visited migrants affected
Orbital ecchymosis	9
Lacerated and bruised wounds	7
Keloids	3
Bone fractures	3
Loss of one or more teeth	3
Extensive burns	2
Cysts	1
Ankylosis of the arm	1

## Discussion

From a demographic point of view, the results obtained are undoubtedly related to the profound social and health changes that have alternated over the years: the annual number of assessments is generally correlated with the number of landings on the Italian coasts, which drastically decreased in 2019 due to government politics, and in 2020 due to the COVID-19 pandemic.

Regarding the sex of the individuals under examination, it also aligns with UNHCR data [5]. The peaks in examinations of self-declared minors from countries in the Indian area in 2020–2021 and from the Maghreb in 2022 appear consistent with the Ministry of Labor data on UFM entries [6]. It is noteworthy that the Ministry data highlights a substantial presence of European migrants in Italy, especially Ukrainians since 2022. However, the latter have never undergone age assessment at the Hospital of Novara, probably because they were received in a more controlled manner by Italy. In 2023, on the other hand, a significant number of examinations were conducted on self-declared minors from Sub-Saharan Africa (64% of the total).

Referring to the situation of UFM in the regional territory, it is possible to note a variation in the methods of requesting assessments over the time. At the beginning, the request almost always came officially from the Prosecutor’s Office at the Juvenile Court of Turin (73% in 2018). Then, the minors’ communities began to directly manage the adolescents hosted, forwarding the request to the Health Direction of the hospital (64% in 2022). In 2023, instead, the social services of the municipality of residence were the primary applicants (78%). There is also a reduction of the number of minors sent for justice needs by the Law Enforcement: this is probably not so much due to a reduction in crimes committed by UFM, but because the presence of forensic pathologists in unscheduled situations cannot be guaranteed in the area. Regarding the pediatric examination, a great operator-dependent variability is highlighted in the reports reviewed, resulting in the absence of a standardized approach in conducting the medical history and physical examination. This was also influenced by the fact that during the COVID-19 pandemic, the procedure was greatly accelerated to avoid prolonged contact with the patient.

More specifically, pediatricians pay great attention to the medical history and general physical examination of the young person, but do not always systematically assess the eruption of the third molar and pubertal development stage. It should also be considered that sometimes the latter aspect may not be evaluated due to the discomfort of the examinee: it was explicitly reported that four male minors opposed the genital inspection.

Regarding the age the forensic pathologist placed the margin of plus or minus 2 years, following scientific evidence and the regional protocol of Piedmont. The ages recorded applying the “-2 years range” were therefore all below 18 years, with an average of 15.81 ± 1.52. The average age obtained by applying the + 2 years range is 19.81 ± 1.52, but this has no legal significance, as for legal purposes the minimum attributable age is considered.

The average difference between the age declared by the examined minors and the certified age is 1.49 years ± 1.52, with a maximum peak of 6 years. The average difference becomes negative considering the minimum attributable age for each minor (applying the − 2 margin): -0.51 years ± 1.52.

Taking into account the margin of uncertainty, in 230 minors (78%) the hospital assessment found an age compatible with the declared one, while in 65 cases (22%) the minimum estimated age was still higher than the declared age and, consequently, incompatible with the latter.

The fundamental and perhaps surprising outcome of this study is the absence of cases where adulthood was confirmed with certainty. The reason lies in the fact that, neither the physical examination with the evaluation of the pubertal development stage, nor the analysis of the wrist X-ray with the Greulich-Pyle method can reveal an age older than 19 years. Therefore, it is mathematically impossible, with the application of the “-2 years margin” suggested by the protocol, to obtain an age over 17 years, and irrefutably ascertain adulthood.

However, it was observed that the UFM included in the study tended to declare a chronological age much lower than the age estimated as most probable from the X-ray examination, and it is difficult to believe that none of them had actually reached the age of 18.

The obtained results were compared with the European literature on the same issue: a German study conducted in Münster in 2020, a Spanish one of 2022 which analyzed the situation in Barcelona and a last one carried out in Montpellier (France) in 2022 [36–38].

The German study [36] included 597 cases evaluated between 2009 and 2018, the Spanish one 2754 cases between 2011 and 2018 [37], and the French one [38] 265 between 2018 and 2021.

In Germany and France, the protocol proposed by the Study Group on Forensic Age Diagnostics (AGFAD), was properly followed, while in Spain a document drafted and shared by all the Spanish Forensic Medical Institutes, then approved by government authorities, was adhered to [39]. The latter permitted the performing of a CT scan of the clavicles in case of discordance between the X-ray and the OPT. In reverse, if these two exams agree on the major age of the examinees, they are immediately considered as adults.

From a demographic point of view, a large majority of male individuals (96.7% in Barcelona, 91.8% in Münster and 97.7% in Montpellier) was also observed in these studies. For obvious geographical reasons, migrations from North African countries (Morocco for 64%) have been massive towards Spain, while a prevalence of Asian migrants (Afghanistan for 33%) was seen towards Germany and a majority of Sub-Saharian people was examinated in France (80.4%).

The reasons for the assessment request observed in the Spanish study seemed more in line with those of the present study (only 3% for justice needs), compared to those included in the German and French analysis, where the judicial authority ordered respectively almost 62% and 80% of the investigations.

Observing the results, it is emphasized that in Barcelona, the majority age was confirmed in 28.8% of the cases, while in Münster 74.5% of the examinees were declared “probably adult”, of which 37.8% “adult beyond any reasonable doubt”. In Montpellier the latter percentage reached 49.4%.

The difference is evidently due to the clavicle CT scan, performed systematically in Germany and France (according to the AGFAD protocol), while only in doubtful cases in Spain.

It should be noted that in Barcelona, the simple result of the wrist X-ray, obtained using the Greulich-Pyle method, confirmed the age of 18 years in about 16% of the cases and 19 years in about 24% (a total of 40.52%). This percentage was far lower than the one found at the Hospital “Maggiore della Carità” (71.5%), but in Spain almost 30% of the examinees were declared adults, while in the Italian sample no one.

In 259 out of 265 individuals examined at the Forensic Medicine Institute of Montpellier, the complete ossification of the radiocarpal joint was observed on X-ray, indicative of an age of 19 years. Nevertheless, it must be considered that with the minimum possible age at this stage is 16.1 years, according to French researchers [14, 15]. This is even lower than the 17 years that would be the Italian result using the empirical “-2 range”. Thus, 246 migrants underwent an OPT (Orthopantomogram), which in 218 cases showed a Mincer stage H (complete mineralization of the apex of the third molar), and in 19 cases the absence of the third molar. However, even the OPT cannot guarantee reaching adulthood: according to Olze’s studies [25, 26], Mincer stage H is compatible with an age of 22.8 years, but the minimum error rate can reach 17.38 years. Therefore, it was necessary to perform a clavicle CT scan on 252 individuals, revealing in 69 cases a Kellinghaus stage 3c, in 52 cases a stage 4, and in 10 cases a stage 5. According to Kellinghaus’s studies [20, 21], the minimum attributable age was 19 years. Therefore, in 131 cases (49.4%), French forensic pathologist certified that the examinee had reached adulthood beyond any reasonable doubt.

Comparing the data from the Novara research, in with the above-mentioned studies, it can be concluded that the 122 UFM diagnosed with an age of 19 years through wrist X-ray would have been eligible to proceed with further assessments. It is reasonable to suppose that, as in the other countries, a portion of them would have undergone to an undeniable certification of adulthood.

From this comparison, it therefore emerges with a good degree of certainty that the main flaw of the Italian local protocol is its inability to answer the primary question posed to the healthcare professionals, which is whether the examinee is certainly an adult or not. It has been seen, in fact, that the answer provided is always negative, and that in similar studies using additional instrumental analysis, this is not always the case.

Among the strengths, the systematic physical examination of the minor allows for direct contact with the young people, helping them understand that the physician is not just an official tasked with discovering their true age, but someone who primarily wants to take care of their health, learn about their conditions, diagnose and treat any possible illnesses. Nevertheless, for the genital examination the ethical problems are not negligible, so that it is expressly discouraged by the European Asylum Support Office [40], and even prohibited by law in other European countries, such as France [41]. Moreover, Tanner himself had expressed doubts about the forensic use of the staging he devised [42]. Furthermore, this study showed that in the cases under review, there is no evident correlation between testicular volume (and consequently Tanner’s stage) and the age estimated as the most probable by the wrist X-ray. Therefore, it can be considered that having the young person undress entirely, given their delicate age and the possibility of past violence, is not essential for age determination. General pubertal development can also be inferred from other factors such as body structure, voice, and body hair (especially axillary hair).

Another strength of the protocol followed at the Novara Hospital is the multidisciplinary nature of the assessment, involving a pediatrician, a radiologist and a forensic pathologist, with the possibility of including a psychologist’s opinion. The contribution of each of these figures is essential to obtain an accurate result, and, compared to other methods used in other Italian areas, the importance of the forensic pathologist is emphasized. Additionally, this is a complex issue that includes numerous laws to observe and involves judicial authorities, which could also ask physicians to justify their decision. Therefore, the presence of an expert in the multidisciplinary team who understands the relationship between medicine and law is further reassuring.

## Conclusion

After reviewing several methods and protocols currently used for age estimation in living individuals, commenting on and comparing the results, it seems evident that age assessment presents several critical issues. For this reason, as widely illustrated, no universally valid protocol has been achieved for its execution. Consequently, individual nations, or even individual authorities, developed and implemented markedly different measures. It has been shown that each method presents intrinsic uncertainty, which, based on the results of this study, can only be overcome through the coordinated use of multiple procedures and the synergy of multiple professional figures.

The effort to assign an age to the self-declared minor as close as possible to the registry one should be seen as a prerequisite for recognizing their real rights. This aspect emerges especially from the comparison with the similar French and German research discussed above: in their Medicolegal Institutes, the protocol proposed by the Study Group on Forensic Age Diagnostic (AGFAD) was systematically followed, allowing for a more precise age diagnosis, considering the uncertainty margins of the individual methods, and overcoming the limitations of a single wrist X-ray.

However, it seems striking that the Italian legislation tends to move away from this model in favor of less invasive, but also significantly less precise methods. The latest national recommendations, in fact, excluded even the figure of an expert such as the Medical Examiner from the team, and indicated the execution of a wrist X-ray only in cases of extreme necessity, considering it as an invasive method, but the subjects included in this study more often refused a thorough physical inspection compared to a radiographic exam, considering the former more invasive of personal intimacy.

It is therefore necessary to address from a bioethical perspective what is the most appropriate approach to obtain concrete and decisive results without violating the human rights and integrity of these individuals.

## Data Availability

The datasets generated during and/or analysed during the current study are available from the corresponding author on reasonable request.

## Footnotes

1 Tanner’s scale was introduced by the British Pediatric Endocrinologist James M. Tanner, who, in relation to the development of primary and secondary sexual characteristics, envisaged five stages of maturation, from 1 (prepubescent) to 5 (adult).
